# Cross-species neuroanatomy in primates using tractography

**DOI:** 10.1007/s00429-025-02914-8

**Published:** 2025-05-28

**Authors:** Stamatios N. Sotiropoulos, Michel Thiebaut de Schotten, Suzanne N. Haber, Stephanie J. Forkel

**Affiliations:** 1 Sir Peter Mansfield Imaging Centre, Mental Health & Clinical Neurosciences, School of Medicine, University of Nottingham, Nottingham, UK; 2 Max Planck Institute for Psycholinguistics, 6525 XD Nijmegen, Netherlands; 3 Brain Connectivity and Behaviour Laboratory, Sorbonne Universities, Paris, France; 4 Groupe d’Imagerie Neurofonctionnelle, Institut Des Maladies Neurodégénératives-UMR 5293, CNRS, CEA, University of Bordeaux, Bordeaux, France; 5 Neurodegenerative Diseases Institute, CNRS, University of Bordeaux, Bordeaux, France; 6 Department of Pharmacology and Physiology, University of Rochester School of Medicine, Rochester, NY USA; 7 Department of Psychiatry, McLean Hospital, Harvard Medical School, Belmont, MA USA; 8 Donders Institute for Brain, Cognition and Behaviour, Radboud University, Nijmegen, Netherlands

## Abstract

Due to their integrative role in brain function, long-range white matter connections exhibit high individual variability, giving rise to personalised brain circuits. This neurovariability is more evident in the connection patterns of brain areas that have evolved more recently. Diffusion MRI tractography allows unique opportunities for comparative neuroanatomy across species to study evolution and provide unique insights into the phylogeny of brain networks, which we overview in this note, inspired by discussions at the International Society for Tractography (IST) retreat.

## Introduction—neurovariability and evolution

Even if long-range white matter (WM) pathways constitute only a small fraction (< 10%) of the total number of neuronal connections in the brain (with the vast majority of connections being intra-cortical) (Schuez and Miller 2002), their integrative role makes them crucial for emerging brain function (Thiebaut de Schotten and Forkel 2022). Function-specific activity involves integrating information from multiple brain regions, and WM connections enable such integration by interconnecting them. WM organisation is complex, and extrinsic connections between regions exhibit high individual variability, which gives rise to personalised brain circuits.

This *neurovariability* provides an anatomical basis for individualised brain function (Saygin et al. 2011) and underlies variability in clinical symptoms and dysfunction due to pathology-induced disruptions in connectivity (Thiebaut de Schotten et al. 2020). There is more to connections than passive transfer of signals, as connections can amplify or reduce information transfer between remote regions. Hence, differences in micro- and macro-structural features of WM bundles across individuals have been associated with differences in response to treatment and recovery (Forkel et al. 2014, 2020).

Neurovariability is further linked to evolution, as brain regions that have undergone recent evolutionary change have higher individual variability in their pattern of connections (Hill et al. 2010; Croxson et al. 2018; Forkel et al. 2022). Concurrently, brain regions that have undergone recent adaptation in humans compared to non-human primates (NHPs) are more susceptible to human-only disorders, such as schizophrenia (van den Heuvel et al. 2019). Hence, mapping connections, their individual variability and their evolution across species can provide great insight into brain function and dysfunction.

Diffusion MRI tractography uniquely allows such mapping of WM connections across individuals (Jbabdi et al. 2015) and species (Assaf et al. 2020). In this note, we overview key efforts of how tractography can be used to perform comparative neuroanatomy between humans and NHPs. In doing so, we also focus on approaches that fuse information across species for augmenting and translating neuroanatomical principles from NHPs to humans, and for discovering modes of success and failure for tractography when comparing to data from invasive neuroanatomy studies in animals, motivating further technical developments.

## Comparative neuroanatomy across species to study evolution

**Fig. 1:**
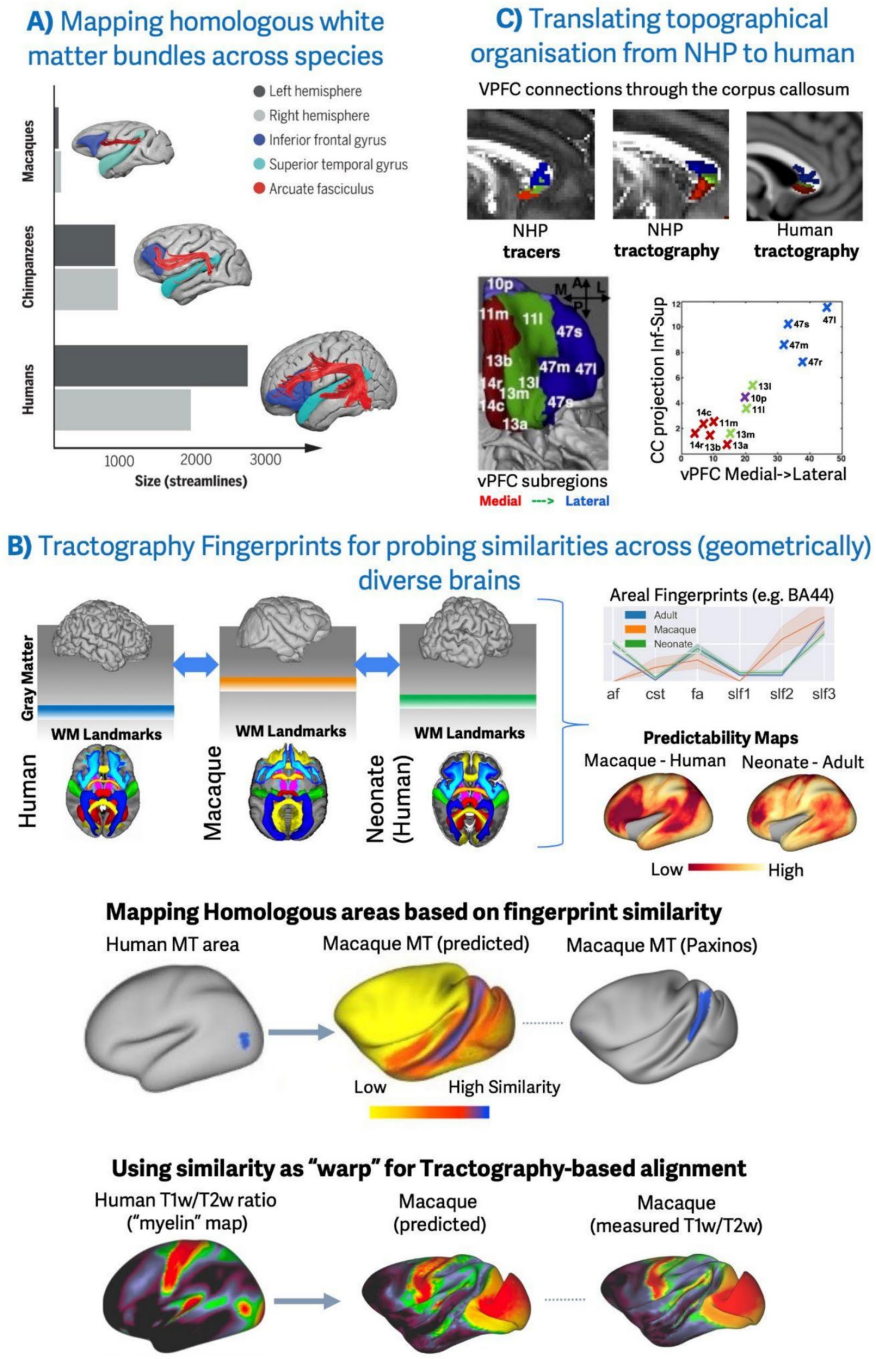
Tractography for cross-species neuroanatomy. **A** Mapping the homologous arcuate fasciculus across 3 primate species reveals a left–right asymmetry in humans that may be linked to language capacity (reproduced with permission from (Balezeau et al. 2020; Thiebaut de Schotten and Forkel 2022)). **B** Tractography fingerprints, i.e. patterns of connectivity of gray matter regions to a set of corresponding WM tracts, can be used to probe similarities and divergences across both phylogeny (e.g. macaque vs human) and ontogeny (e.g. neonate vs adult) of brain connectional organisation. Using tractography, corresponding WM tracts across diverse brains can be mapped and act as landmarks, upon which a latent common connectivity space can be defined. Tractography fingerprints can then be obtained as the patterns of connections of brain regions with respect to these WM landmarks (e.g. visual areas will be more connected to optic tracts). They can be constructed as (GM locations/regions x WM tracts) matrices, with *n*^*th*^ row corresponding to the tractography fingerprint of the *n*^*th*^ cortical location/region (blue, orange, green rows in 3 diverse brains in top of panel B) and the *m*^*th*^ column corresponding to *m*^*th*^ WM bundle. Similarity in such patterns can be used to produce predictability maps between diverse brains (top of panel B), to map homologous areas (middle row of panel B, predicting the location of macaque MT based on similarity to connection patterns of human MT), and as a warp field to drive tractography-based alignment (bottom row of panel B, aligning a “myelin” cortical map from human to macaque, using the similarity of areal tractography patterns between human and macaque as a (geometry-free) warp field). Figures modified from (Mars et al. 2018b; Warrington et al. 2022). **C** Learning topographical organisation principles of connections from NHP tracers and translating them with tractography to humans. Tracer injections from the medial to the lateral ventral prefrontal cortex (vPFC) of the NHP brain show connections organised topographically from inferior to superior in the genu of the corpus callosum. This pattern can be recovered using NHP tractography and translated to human tractography (modified with permission from (Jbabdi et al. 2013))

Most of our knowledge of primate neuroanatomy comes from tracer injection studies in animals, with the monkey brain being commonly used as a model of the human brain. Therefore, comparative studies of brain connections are essential, as they link findings from non-human primate (NHP) studies to human anatomy and allow us to study evolutionary trajectories. Tractography provides unique possibilities for identifying and mapping *homologous white matter tracts across species*. For instance, Rilling et al. (2008) used diffusion MRI data from the macaque monkey and the human brain and found that the human arcuate fasciculus has a prominent temporal lobe projection, which is much smaller or absent in NHPs. Balezeau et al. (2020) used tractography in the macaque, chimpanzee and human brain to map bilaterally the arcuate fasciculus, and proposed that its remarkable left–right asymmetry, identified only in humans, is linked to language capacity (Fig. 1A). Barrett et al. (2020) used tractography to reconstruct a number of homologous tracts in humans and three monkey species and showed that humans have a greater proportion of frontal lobe connections, when normalised by total white matter volume.

Tractography can also be used to identify *homologous cortical areas across species* based on the similarity of their areal connectional patterns (Mars et al. 2018b) (Fig. 1B). The pattern of extrinsic connections of a brain region to other regions or WM bundles is unique and linked to its function (Passingham et al. 2002) (e.g., the primary visual cortex is more connected to the optic radiation, the inferior fronto-occipital fasciculus, the ventral occipital fasciculus and not connected to the arcuate fasciculus, the frontal aslant tract and the genu of the corpus callosum). Tractography can provide a proxy to these connectional patterns or *tractography fingerprints* of brain regions with respect to a set of WM bundles (Fig. 1B—top row). If the same set of corresponding WM bundles are reconstructed across different species, the tractography fingerprints of regions can be compared across species (e.g. blue, orange and green rows in Fig. 1B—top) and homologous brain regions can be identified on the basis of tractography fingerprint similarity (Mars et al. 2018a, 2021). A number of studies have proposed tractography protocols for reconstructing corresponding WM bundles whose central cores are known to exist in both humans and NHPs, for instance, in macaques and humans (Warrington et al. 2020; Assimopoulos et al. 2024), chimpanzees and humans (Bryant et al. 2020, 2025) or great apes and humans (Roumazeilles et al. 2020). Tractography fingerprints can then be estimated for all regions with respect to this same set of WM bundles, providing a common space for performing comparative comparisons (Mars et al. 2018b).

The similarity of tractography fingerprints can be used across species to identify whole-brain convergences (e.g. homologous areas across species) and divergences (e.g. areas that have undergone recent evolutionary change) (Fig. 1B—top, middle row). It can even be used as a geometry-free warp field to translate scalar maps across species (Mars et al. 2018b) (Fig. 1B—bottom row). Same principles can be extended more generally across geometrically-diverse brains, such as the adult and neonatal human brain, enabling to map evolution concurrently with neurodevelopment timescales of brain connectivity (Warrington et al. 2022). For instance, the predictability maps in Fig. 1B (top row) indicate areas with tractography fingerprints that have undergone recent evolutionary change (depicted in dark red on the left) and areas with fingerprints that develop later in humans (in dark red on the right). These technical advances enable targeted studies to improve animal models of human disease and translate mechanistic discoveries from animals to humans (Thiebaut de Schotten et al. 2014).

## Learning and translating white matter organisation principles from NHP to human

Tractography approaches can be combined with classical neuroanatomy tools, such as chemical tracing or dissections, to learn and translate principles of white matter organisation from animals to humans. Catani et al. (2017) devised tractography protocols for reconstructing parietal bundles in the macaque and the human brain and used dissections to guide and validate these protocols. Thiebaut de Schotten et al. (2011) utilised evidence from tracers in monkeys to develop human tractography protocols to reconstruct different segments of fronto-parietal connections. These were subsequently used to study the anatomical basis of visuospatial attention. In (Jbabdi et al. 2013) chemical tracers injected at different parts of the ventral prefrontal cortex revealed a topographical organisation of vPFC connections through the genu of the corpus callosum and the internal capsule. Medial vPFC projected through inferior callosal regions, while lateral vPFC projects to more superior callosal regions (Fig. 1C). These topographies were reconstructed using NHP tractography and translated to human tractography (Jbabdi et al. 2013; Sotiropoulos et al. 2016), where chemical tracing is unavailable.

Similar topographic organisation revealed by tracers and confirmed with NHP tractography has allowed the parcellation of the human anterior limb of the internal capsule into distinct sub-segments that carry fibres from different prefrontal cortex areas (Safadi et al. 2018). Several other studies have not relied on topographies but on translating connectivity patterns in the macaque brain (known from tracers) to in-vivo tractography in humans. For instance, Rushworth et al. (2006) have distinguished three regions in the human parietal cortex using tractography and replicating patterns of connections known in the NHP brain. Similar ideas have been applied to the temporo-parietal junction (Mars et al. 2013) and the dorsal frontal cortex (Sallet et al. 2013). The translation of such principles and neuroanatomical patterns can be important for understanding pathology in humans by associating disease abnormalities in these pathways with specific connections, but also informing targets for intervention and treatment (Haber et al. 2021), (Haber et al. 2023).

## Validation of tractography in the primate brain

While enabling non-invasive, whole-brain and at scale explorations, accuracy of dMRI tractography can be a challenge due to its indirect and macroscopic nature (Reveley et al. 2015; Jbabdi et al. 2015; Sotiropoulos and Zalesky 2019; Maier-Hein et al. 2019). Tractography approaches can benefit from prior anatomical knowledge and guidance (Warrington et al. 2020; Schilling et al. 2020), which can be obtained through different modalities in the primate brain. Connectional anatomy of the NHP brain—see (Yendiki et al. 2022) for a review—provides a testbed for validating and optimising tractography algorithms and protocols applicable to the human brain.

Specifically, the plethora of NHP data available from complementary invasive imaging methods (chemical tracers, microscopy, histology) can be utilised, integrated with and compared against tractography estimates mapped from diffusion MRI. At the same time, post-mortem MRI allows for longer and higher-resolution acquisitions. Even if the rodent brain provides opportunities for similar validation studies (Oh et al. 2014), the neocortex forms just 28% of the brain in the rat, compared to 72% in the macaque monkey (Passingham 2009) [much closer to the 80% in the human brain (Kaas 2019)], making neuroanatomical comparisons in the NHP brain more translatable to human.

Several studies have utilised data from different modalities acquired on different brains to perform comparisons (for instance, microscopy data and diffusion MRI data acquired from different sets of animals, respectively). This is sometimes necessitated by the nature of the aimed task and the data at hand. For instance, Donahue et al. (2016) assessed the efficacy of tractography in predicting whole-brain connectivity patterns in the macaque by comparing against connectomes weighted by the fraction of labelled neurons, obtained from retrograde tracer injections in tens of different animals (Markov et al. 2014). Girard et al. (2020) followed a similar paradigm, comparing tractography estimates against binary connectivity matrices from tracers.

More recent studies have acquired multi-modal data on the same NHP brains. In (Safadi et al. 2018), the same animals received tracer injections and post-mortem dMRI. The tracers revealed a topographic organisation of fibres in the anterior limb of the internal capsule, which was successfully reproduced by tractography. A similar set of within-animal experiments is presented in (Grisot et al. 2021) to identify modes of failure of tractography, particularly in cases of fibre branching or sharp turning, as in (Maffei et al. 2022).

Due to the power of having multiple modalities in the same brain for tractography validation and development, recent efforts have focused on building much-needed data resources. Howard et al. (2023) put together an extensive dataset that combines data from four orders of magnitude of spatial resolution in the same NHP brain, ranging from in-vivo and ex-vivo MRI, to polarised light imaging and myelin-stained histology. Several recently launched large-scale efforts follow the same paradigm to provide high-quality MRI data along with 3D microscopy modalities and tracers in the same primate brains (e.g. LINC—https://connects.mgh.harvard.edu/, CMC—https://www.mesoscale-connectivity.org). Openly accessible resources from these consortia and others (such as PRIME-DE (Milham et al. 2018) or the BRAIN/MINDs portal (Hata et al. 2023), which also offer quality assurance protocols) will be invaluable in developing the next generation of tractography approaches, where a transfer learning paradigm from microscopy to MRI (Zhu et al. 2025) could assist in optimising and improving specificity and sensitivity of tractography methods.

## Data Availability

No datasets were generated or analysed during the current study.
